# New proteomic signatures in Alzheimer Disease identification and Mild Cognitive Impairment Classification

**DOI:** 10.1002/alz.094836

**Published:** 2025-01-09

**Authors:** Manyue Hu, Oliver J Robinson, Christina C Lill, Anna Matton, Raquel Puerta, Agustin Ruiz, Lefkos T Middleton

**Affiliations:** ^1^ The Ageing Epidemiology (AGE) Research Unit, School of Public Health, Imperial College London, London, England United Kingdom; ^2^ Imperial College London, London, England United Kingdom; ^3^ Imperial College London, London United Kingdom; ^4^ Ace Alzheimer Center Barcelona – International University of Catalunya (UIC), Barcelona Spain; ^5^ Research Center and Memory Clinic, Fundació ACE Institut Català de Neurociències Aplicades ‐ Universitat Internacional de Catalunya (UIC), Barcelona Spain; ^6^ The Ageing Epidemiology (AGE) Research Unit, School of Public Health, Imperial College London, London United Kingdom

## Abstract

**Background:**

Emerging technologies and novel biomarker tools are transforming the field of Alzheimer’s disease, allowing for a more in‐depth exploration of biological mechanisms underpinning the disease aetio‐pathogenesis. In this context, there is growing recognition of the potential of plasma proteomics for AD risk assessment and disease characterization. On the other hand, differences between proteomics platforms introduce uncertainties regarding cross‐platform applicability.

**Method:**

Elastic net analysis was applied to 190 analytes measured by the Luminex xMAP platform in plasma from 566 participants [58 cognitively normal (CN), 396 with mild cognitive impairment (MCI) and 58 with dementia due to AD], from the Alzheimer’s Disease Neuroimaging Initiative (ADNI). Validation was conducted on 1303 participants (113 CN, 795 MCI, 395 AD) from the Spanish ACE study, which was analysed by the SOMAscan 7k proteomic platform. MCI participants were classified into MCI stable or decliners according to their follow‐up diagnosis (ADNI: 158 vs. 217, Ace: 449 vs. 346). Signatures derived from our elastic net analysis and other methods applied in prior ADNI proteomic signature studies were compared, with our approach aimed at reducing dimensionality of signatures of AD vs. CN or MCI subgroup classification.

**Result:**

We identified a signature including 11 plasma analytes and sex, age, and APOEε4 gene status, for distinguishing AD from CN, with 94% accuracy on ADNI (sensitivity 88%, specificity 94%) and 95% on Ace (specificity 96%, sensitivity 93%). Another 19‐analyte signature with the above confounders was used for classifying MCI stable and decliners, with a 77% accuracy on ADNI (sensitivity 85%, specificity 40%) and 51% on Ace (sensitivity 68%, specificity 36%). These two signatures share one common analyte, Vitronectin, a protein found to appear in degenerative diseases related abnormal deposits, such as amyloid.

**Conclusion:**

Compared with prior proteomic signature studies, all based on ADNI, our findings attained higher specificity and sensitivity, while utilizing a smaller panel for AD classification. They also confirm the reliability and consistency of a group of plasma proteomic biomarkers across two different platforms.

